# Role models as a factor influencing career choice among female surgical residents in Saudi Arabia: a cross-sectional study

**DOI:** 10.1186/s12909-022-03181-5

**Published:** 2022-02-19

**Authors:** Rawan Abdulrahman T. Harun, Reem Almustafa, Zainab AlKhalifah, Abdullah Nammazi, Abdalmohsen AlBaqami, Nourah Mohammed ALSaleh, Mai Kadi, Ali Farsi, Nadim Malibary

**Affiliations:** 1 Faculty of Medicine, King Abdulaziz University, Jeddah, Saudi Arabia; 2 Department of General Surgery, Al-Noor Specialist Hospital, Makkah, Saudi Arabia; 3 Department of Surgery, King Abdulaziz University, Jeddah, Saudi Arabia; 4 Department of Community Medicine, King Abdulaziz University, Jeddah, Saudi Arabia

**Keywords:** Role model, Female surgeons, Career choice, General surgery

## Abstract

**Background and purpose:**

Role models in the medical field are professional and experienced persons whose actions unconsciously inspire juniors to strive to be like them. To our knowledge, no studies have examined whether having a female surgical role model has influenced women to pursue a surgical career in Saudi Arabia. Hence, we sought to evaluate whether identifying role models in surgery influences career choice and defined the ideal qualities of a surgical role model as perceived by newly qualified doctors.

**Methods:**

We employed a cross-sectional, survey- based study design, conducted between June 2020 and January 2021, in which female surgical residents completed a questionnaire about their perceptions and influence of role models in general surgery specialty at the time they pursue a career in surgery.

**Results:**

A total of 51 respondents completed the questionnaire. The majority of them (78.4%) had a role model and (19.6%) indicated that their role model was a female. Of those who had a role model, (67.5%) agreed that they experienced a positive influence on their surgical career choice. Clinical and operation skills were reported as the most remarkable factor to be considered in their role models. Working directly with a male surgical consultant and female surgical residents imprinted a positive influence but this did not reach a statistical significance.

**Conclusion:**

The findings of this article contribute empirically to the strong effects of the same-sex role models and highlight the curial role of surgical residents in influencing female’s career choices in general surgery as an achievable and attainable domain. Which encourage the creation of national mentorship programs and exploring more in barriers for pursuing a surgical career.

## Introduction

Every year, women represent an ever increasing proportion of students admitted to medical school [1]. In some institutions, women now outnumber men, comprising more than 50% of medical classes [1, 2]. Nevertheless, in surgical programs, there remains a huge enrollment difference between genders [3].

A study conducted in nine medical schools in the United States (US) documented that whereas 24% of men showed an interest in surgery, only 15% of women did so [4]. As 2020, only 13% of consultant surgeons in England were women, at the same time they reported a slow increasing of female registers in surgical training [5]. Other studies in the US report a steady rise in the number of women pursuing surgical, leading to questions regarding the cause of this attraction to surgery as a career [6]. Consequently, an extensive amount of literature exists exploring factors that influence women when considering a surgical career, including enjoyment of surgical clerkship, future income and opportunities, academic interest, hands-on-work, prestige, and the impact of access to role models in gender-based schooling and occupational segregation [7–9]. Role models in the medical field are professional and experienced persons whose actions unconsciously inspire juniors to strive to be like them [10]. A systematic review [11] has extensively explored the factors affecting female surgeons in choosing their career; the evidence in local studies is, however, insufficient [12]. In a study conducted in Japan, female surgeons described the importance of having a role model to help them be more productive in their careers, although they indicated that finding the ideal model was difficult [13]. Another study in Virginia revealed that 35% of female medical students were discouraged from pursuing a surgical career because of the lack of female role models [4]. The presence of a female role model is therefore essential in inspiring women to pursue a surgical career by making it more “real and attainable” [8, 14].

Previous studies in the Kingdom of Saudi Arabia (KSA) endeavored to investigate medical students’ specialty preferences and influential motives towards selecting a prospective specialty. A survey of all medical students in their basic and clinical years reported that females who chose surgery as their future career were ranged between 16.28%—20.8% while males were 26.3%-33.1% [7, 15]. To our best knowledge no studies in KSA have assessed the impact of role models on the career choice of female surgeons. Therefore, in this study we aimed to investigate role models and the presence of female surgeons in the field as influencing factors for aspiring female surgeons in KSA.

## Methods

### Study design, setting, and time frame

A cross-sectional study was performed at the Department of Surgery of King Abdulaziz University Hospital, a tertiary center in Jeddah, Saudi Arabia. The study took place between June 2020 and January 2021.

### Study participants

All female general surgery resident who are currently enrolled in the general surgery residency program in Jeddah were contacted to fill out the study survey.

### Sample size and sampling procedure

There are 11 residency training centers in Jeddah, which has a papulation of 65 female surgical residents. The surveys were sent to be randomly filled out by respondent. The response rate was 93.8% (*n* = 61). Ten responses were eliminated due to missingness. Therefore, we included 51 in the final analysis.

### Data collection instrument

We used Google forms to develop an anonymous questionnaire containing 31 items to be sent by the surgery program directors to all female surgical residents who registered via the Saudi Commission for Health Specialties (SCFHS) across Jeddah, Saudi Arabia. The questionnaire was based on a previously validated questionnaire used by O’Herrin et al. [16]. Further, new questions about the effect of role models and the presence of female surgeons and their influence on female students in pursuing a surgical career were added after an extensive literature review [15, 17–19]. Four independent experienced surgeons with a background in teaching residents further validated the modified questionnaire, and their suggestions were incorporated.

The survey included four sections. The first section addressed participant demographics, current level of training, and first choice of residency program. The next section included items designed to assess the effect of family on career choice rated on a 5-point Likert-scale ranging from 1 (no influence) to 5 (strong influence). For example, one question read, “How strongly did your relationship with your spouse influence your decision to pursue a surgical career?” The third section focused on the importance of role models. The definition of role model was presented as follows: “Role model: Is a professional and experienced person whose actions unconsciously inspire juniors to strive to be like him/her.” The purpose of this section was for the participant to evaluate the definition and character of a surgical role model and the influence of that person on the respondent’s career choice. This section included questions about the gender of the role model and qualities that the participant looked up to in the role model (compassion, skills, respect, knowledge, integrity, and others). Respondents were also asked whether they felt that female surgeons were well represented in our society and if this affected their choice of surgery. The fourth section included items to assess factors that influenced the respondent’s choice of surgery as a career. The interpretation of the factors was left to the individual and no definition of any factor was provided.

### Data analysis

Data were analyzed by using the SPSS software program (version 24). Qualitative data are expressed as frequencies and percentages and quantitative data as means and standard deviations. The chi-square test was used to determine the relationship between variables. A *p*-value of < 0.05 was considered statistically significant.

### Research ethics

The study was approved by the Institutional Review Board of KAUH (Reference no.: 62–20). The study was conducted in compliance with the requirement of the Declaration of Helsinki. All medical resident participants gave written informed consent at the beginning of the study.

## Results

Of the 65 female surgical residents, 51 completed the questionnaire (78.5% response rate). The mean age of the participants was 27.37 ± 2.13 years, and 41.2% were in their first year of the residency. For 70.6% of the respondents, GS was the first choice when they applied for residency training. 3.9% of the participants were married at the time of the survey. Notably, female surgical residents rarely cited the childcare and their spouse relationship as strong influential factors in their decisions when they pursuit a career in surgery, 16% and 7.8% respectively. Eighty-two percent reported that teaching is important to their career choice. Further demographic data on the survey respondents are summarized in Table 1.

**Table 1:** Distribution of studied participants according to their characters, training level and conditions related to choosing general surgery as a career

Variable	No. (%)
Age (year)	27.37 ± 2.13
Nationality	
Non-Saudi	5 (9.8)
Saudi	46 (90.2)
What is your current level of training?	
PGY 1	21 (41.2)
PGY 2	5 (9.8)
PGY 3	8 (15.7)
PGY 4	9 (17.6)
PGY 5	7 (13.7)
Service resident	1 (2)
Was general surgery your first choice when applying for residency?	
No	15 (29.4)
Yes	36 (70.6)
Marital status when you decided to pursue general surgery	
Engaged	1 (2)
Married	2 (3.9)
Single	48 (94.1)
With whom do you live currently?	
Alone	7 (13.7)
With family	44 (86.3)
Did you have children when you applied for general surgery?	
No	50 (98)
Yes	1 (2)
How many children did you have?	
0	50 (98)
2	1 (2)
Do you think, in general, that childcare influences your career choice?	
Neutral	17 (33.3)
Not at all	9 (17.6)
Not very strongly	9 (17.6)
Somewhat strongly	8 (15.7)
Very strongly	8 (15.7)
How strongly did your relationship with your spouse influence your decision to pursue a surgical career?	
Neutral	8 (15.7)
Not applicable	26 (51)
Not at all	8 (15.7)
Not very strongly	2 (3.9)
Somewhat strongly	3 (5.9)
Very strongly	4 (7.8)
Is teaching important to your career choice?	
No	9 (17.6)
Yes	42 (82.4)

*Abbreviations*: *PGY* postgraduate year

Fourty residents (78.4%) of the participants indicated that they had a role model, 31.4% of them had both genders as a role model, and 54.9% had chosen that person as a role model during residency training. More than half of the participants (67.5%) reported that the role model positively influenced their choice at the time they pursue a career in surgery, and 68.6% reported that the role model's practice was in an academic/university hospital setting.

**Fig. 1:**
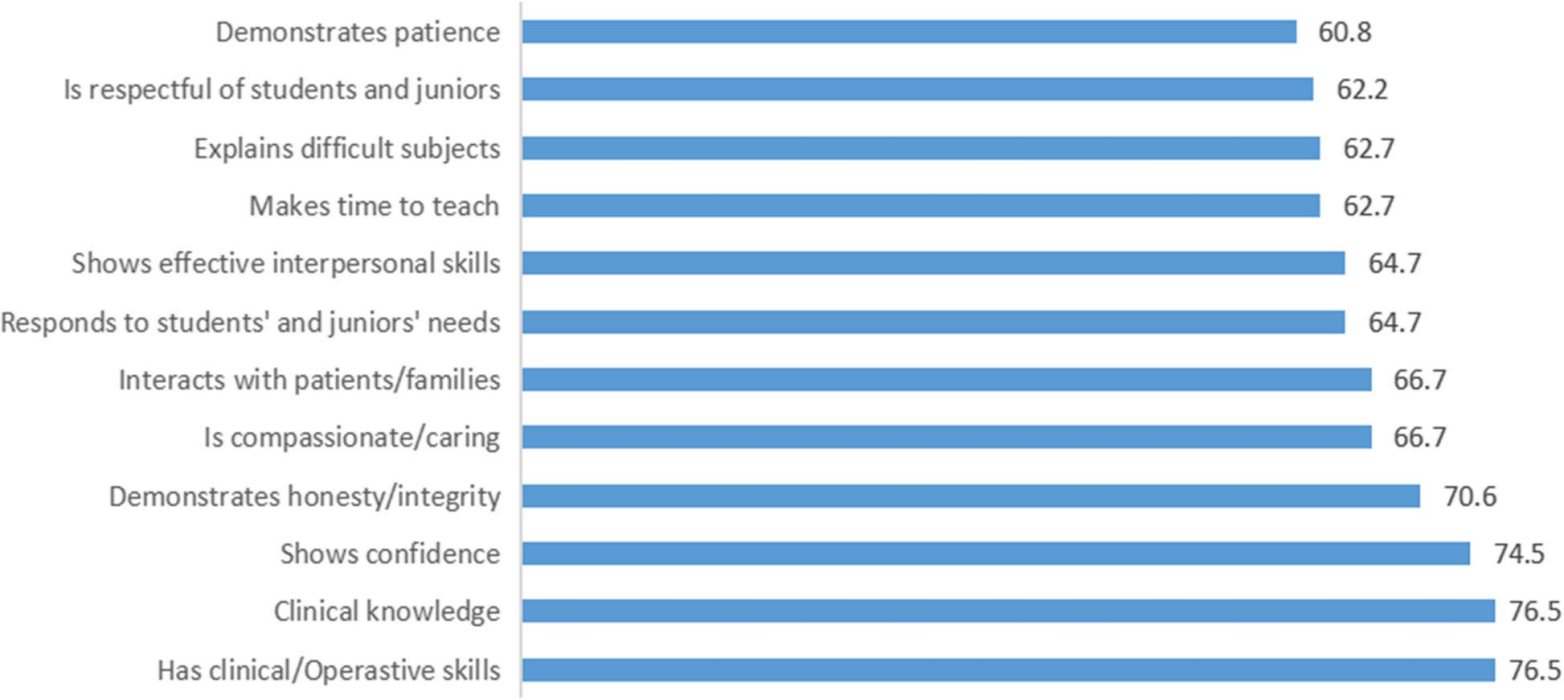
Participants’ mean ratings of specific attributes important in selecting a surgeon role model

Figure 1 shows; clinical/operation skills, clinical knowledge and show confidence were the most chosen role model’s characters among female residents surgeons.

The survey included two questions regarding the influence of a female and male surgeons’ presence in the field. “Do you think that the presence of a female\male surgeon in your clinical training affected your decision at the time you pursued a career in surgery” which was a yes, positively or yes, negatively or no, it did not question. 41.2% responded “yes, positively” that female surgeon influenced their decision at the time they pursuit a career in surgery, while 76.5% agreed on that the presence of male surgeon in their clinical training program did not influence their decision.

Table 2: shows most participants (68.6%) discussed a surgical career with a female surgeon before applying to surgery, 92.2% worked with a female surgeon before applying to surgery, and 35.3% thought that female surgeons are properly represented in their community.

**Table 2:** Distribution of studied participants according to the effect of the surgeon's gender on participants’ career

Variable	No. (%)
Do you think that the presence of a *female* surgeon in your clinical training affected your decision at the time you pursued a career in surgery?	
No, it did not	30 (58.8)
Yes, negatively	0 (0.0)
Yes, positively	21 (41.2)
Do you think that the presence of a *male* surgeon in your clinical training affected your decision at the time you pursued a career in surgery?	
No, it did not	39 (76.5)
Yes, negatively	2 (3.9)
Yes, positively	10 (19.6)
Had you discussed a surgical career with a female surgeon before applying to surgery?	
No	16 (31.4)
Yes	35 (68.6)
Had you worked with a female surgeon before applying to surgery?	
No	4 (7.8)
Yes	47 (92.2)
Do you think female surgeons are properly represented in your community?	
No	33 (64.7)
Yes	18 (35.3)
Were your choices influenced by a female surgical resident?	
No	27 (52.9)
Yes, negative influence	1 (2)
Yes, positive influence	23 (45.1)
Have you worked directly with her?	
No	2 (3.9)
Not applicable	17 (33.3)
Yes, after applying	2 (3.9)
Yes, before applying	30 (58.8)
Were your choices influenced by a female surgery consultant?	
No	26 (51)
Yes, positive influence	25 (49)
Have you worked directly with her?	
No	7 (13.7)
Not applicable	16 (31.4)
Yes, after applying	3 (5.9)
Yes, before applying	25 (49)
Were your choices influenced by a male surgical resident?	
No	31 (60.8)
Yes, negative influence	2 (3.9)
Yes, positive influence	18 (35.3)
Have you worked directly with him?	
No	1 (2)
Not applicable	20 (39.2)
Yes, after applying	2 (3.9)
Yes, before applying	28 (54.9)
Were your choices influenced by a male surgery consultant?	
No	25 (49)
Yes, positive influence	26 (51)
Have you worked directly with him?	
No	1 (2)
Not applicable	19 (37.3)
Yes, after applying	2 (3.9)
Yes, before applying	29 (56.9)

**Fig. 2:**
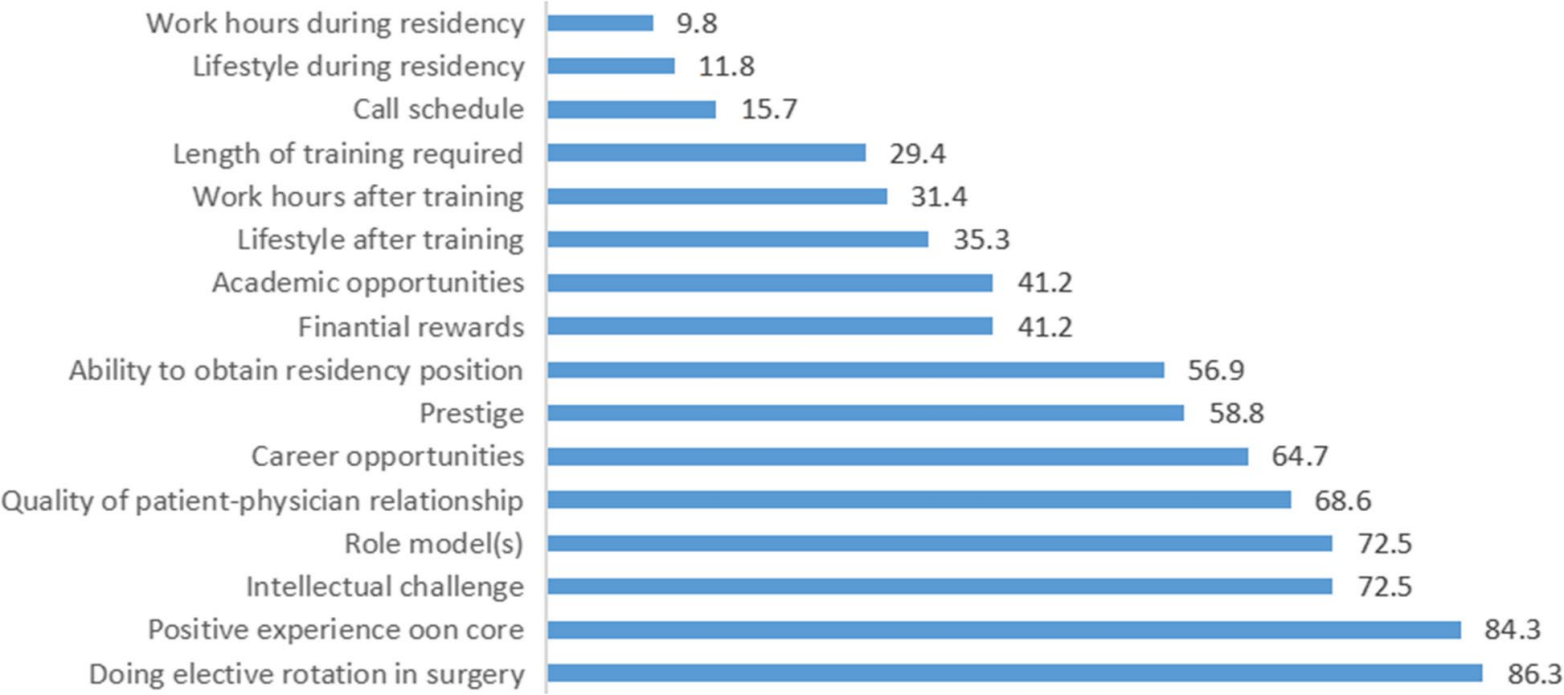
The respondents’ perceived influential factors in choosing a career in surgical field

Figure 2 shows the most common picked factors that positively influenced the participants’ decision to pursue a surgical career: doing an elective rotation in surgery (86.3%), positive experience on the core rotation/internship (84.3%), role model(s) and intellectual challenge (72.5%), quality of patient-physician relationship (68.6%), and career opportunities (64.7%). A nonsignificant relationship was found between having a role model and GS being the first choice when applying to residency (*p* > 0.05).

**Fig. 3:**
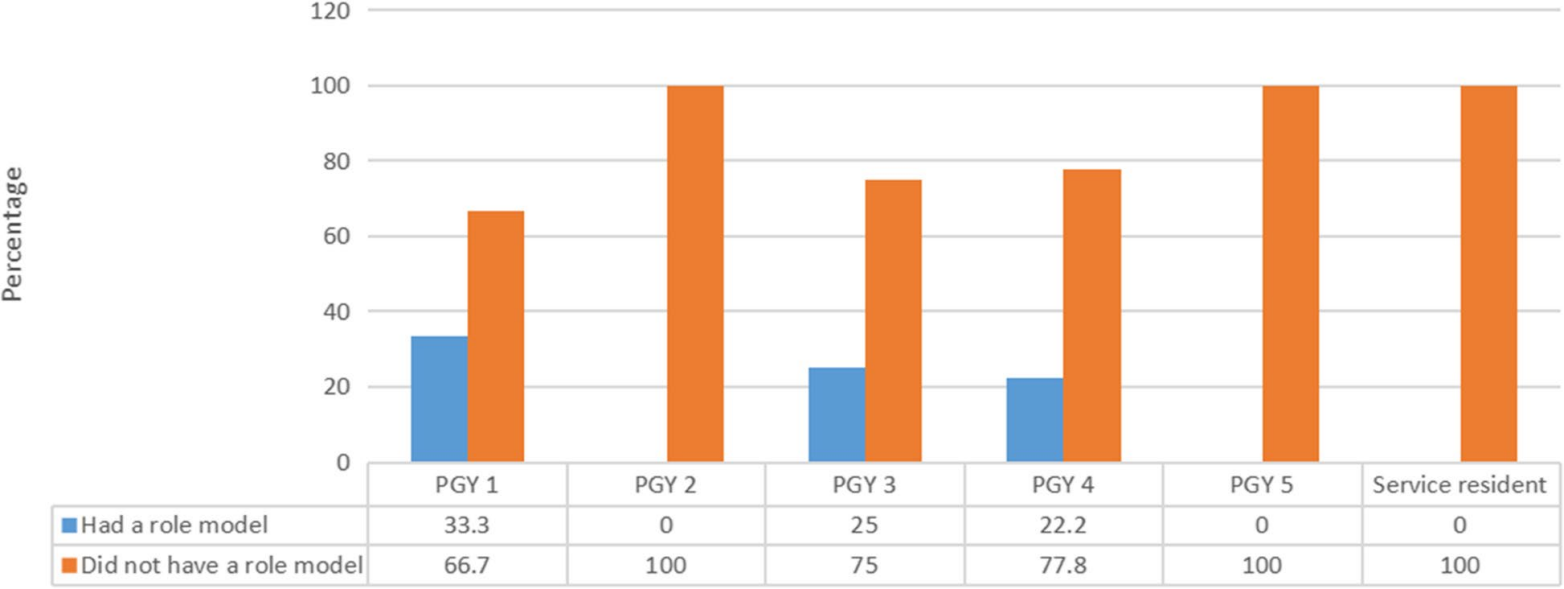
Relationship between having a role model and current level of training

Figure 3 illustrates that a nonsignificant relation was found between the level of training and having a role model (*p* > 0.05).

As well as our results shows that a non-significant relationship of participants who worked directly with a female or male surgery consultant before applying reported that their role model positively influenced their choice at the time they pursued a career in surgery (*p* > 0.05). Furthermore, a nonsignificant relationship was found between the effect of the role model at the time of pursuing a career in surgery and all other effects of the surgeon’s gender on the participants’ careers (*p* > 0.05).

## Discussion

Our goal in this study was to identify the effect of a role model in general surgery, we also wanted to assess the impact of the presence of female surgeons in the field. Our results revealed several interesting findings, the majority of female surgical residents had a role model, half of them, sixty-seven percent reported that the role model, regardless of their gender, have a positive influence on them at the level of specialty decision making. Moreover, female surgical residents provide preliminary evidence that the presence of female surgeons in the field and working directly with female residents and male consultants before applying to general surgery residency program significantly influenced their choices at that time. A further novel finding is that female surgical residents rarely cited that family obligations as strong influential factors in their decisions when they.

### Role model

Several factors influence a female surgeon's decision to pursue surgical training. Among these are surgical clerkship experiences and interactions with attending physicians and residents [16]. However, few literatures have studied the association between exposure to role models and decision to pursue a career in surgery [13, 20]. It has been demonstrated that the influence of role models is a significant factor determining the choice of postgraduate training among medical students [8, 21–24]. Most of our study population (78.5%) had a role model, similar to the results of an earlier study in which 68.6% of female students reported that the presence of role models was the top factor in choosing a career in surgery [23]. This is important as female physicians who have a mentor are more motivated to write articles and participate in research and to show greater career satisfaction [25]. These findings highlight the formative influence of a role model in shaping a physician's professional identity, in particular at the level of specialty decision making.

In the eyes of the residents, clinical knowledge, operative skills and demonstrate integrity and honestly and making time to teach are the most important characteristics in a role model. This finding is comparable with that reported by Wright et al. [26] in which clinical skills were also ranked highly. When we inquired about the role models' teaching skills, this response supports the idea that university hospitals may be the ideal setting for medical role models in Saudi Arabia. In contrast to the results of our study, the sample in a study by Sternszus et al. [18] did not assign as much importance to clinical skills. One explanation for this discrepancy may be that in Saudi Arabian society, competition for general surgery residency spots in the most prestigious centers is high, leading trainees to value the clinical practice skills that they can acquire from a surgeon above all else. In addition to that, trainees may also value more humanistic qualities, including adequate interaction with patients and their families, honesty and integrity, and ethical behavior. Research in positive psychology and moral philosophy suggests that "moral elevation," the experience of positive moral emotions after witnessing an exceptional attitude from a role model, may sustain the influence of role models on the choice of specialty [27].

### Family and lifestyle

Female surgeons placed minimal emphasis on the influence of childcare and their spouse on their decision to pursue a career in GS. Interestingly, it is commonly assumed that these factors are more influential for women. For example, Sanfey et al. [4] indicated in their study that concerns about combining a surgical career and surgical training with future lifestyle, family life, and parental responsibilities were more significant for women than for men. On the other hand, some studies suggest that these gender differences are no longer as evident [17, 28]. They reported a tendency for both men and women to agree on issues regarding family or lifestyle priorities when considering the pursuit of a surgical career. In another study, more men than women chose practice lifestyle as influencing their career choice [26]. Of our 51 respondents, only 3.9% were married, most being single, as observed in other studies [29, 30]. Therefore, it can be assumed that both the demanding nature of surgery and the length of the residency period make it difficult for women to achieve a balance between their surgical career and family life. Another thing to be considered, It was often reported that women prioritise career advancement by delaying starting a family. Female surgeons in the US were twice as likely as male surgeons to delay having children until after postgraduate residency training; women who had children early in their career felt less financially secure and more anxious than women who commenced families later in their career [31]. Work hours and residency lifestyle may also be attributed to women’s shifting interest toward medical specialties with more flexible lifestyles that are more suitable to the current generation [32].

### Presence of a female surgeons

Another area that has been little analyzed in the literature is the impact of the presence of female surgeons on the number of female surgical residents. This number has been slowly growing with each year. Our results show 41.2% of female surgeon residents who pursuit a career in surgery were affected by presence of female surgeons in the field. Some authors have speculated that women may view a specialty as being more attainable and achievable if they see another woman in the same position [33]. Other studies have shown similar trends of women choosing surgery as a career when there is a high proportion of female faculty members and female residents [14, 34]. Organizational and social psychological research suggests that women might be subjected to greater scrutiny because they are pressured to represent their group, i.e., other women. Furthermore, women face more stereotypical threats in comparison to men. Therefore, women fear being judged more than men do. If that is the case, a more well-balanced gender composition in such a setting may increase women’s self-confidence, encouraging them to take part [35, 36].

### Working with seniors in the field

Interaction with residents was the most common factor influencing the choice of surgery as a career [25]. In addition, our results show that women are more likely to be influenced by other female residents or surgical consultants if they worked directly with them. Interestingly, however, the overall influence of a female resident was more than that of a consultant. This trend also applied to male residents and consultants, but in reverse, with male consultants being more influential than male residents. Regarding differences between consultants and residents, other studies have also shown various patterns. One reported that consultant role models are more important than resident role models [18]. This may be because consultants have overall more experience and skills in the surgical field. Other studies claim that residents outscored attending physicians in the eyes of students [37, 38].

We hypothesis that since surgical fields have traditionally been a male-dominated profession, it is not surprising to have male consultants have an influence in female residents more than female consultants did. With time the increased enrollment of female medical students in GS, increased the number of female surgical residents, that being said residents are usually more approachable and thus have an excellent positive influence on younger students. Another hypothesis is that residents are nearer in age to younger students, bringing their teaching methods and skills closer to the students’ level. This opens room for future application of peer training in addition to staff-led training, which may elevate the overall level of the learning experience.

### Other influential factors

In exploring the effect of other factors besides role models, our results show that a positive experience in core rotations highly encouraged residents to choose GS. It has been suggested that clerkship offers interaction with senior residents and attending surgeons who may serve as potential positive future role models [39]. The study by Berman et al. [39] supported this possibility when they found that surgical clerkship plays a large role in influencing career choice, possibly because actively participating in the operating room and seeing the specialty up close makes students feel more involved.

Our study also shows that work hours and residency lifestyle are the most negative influencing factors. These findings are similar to those from American studies in which work hours and lifestyle were chosen as factors that would discourage one from pursuing GS [4, 40].

There are some limitations to this study. These findings are the opinions of the female surgical residents in Jeddah, KSA and opinions may vary geographically. So, we suggest undertaking studies on a larger scale by repeating the study on a larger group with various backgrounds and in different countries to extend these findings to eliminate selection bias. Secondly, this study has established the importance of surgical role models, but there is further work to be done as to how this can be positively used by the profession to encourage young women to embark on and persist with a surgical career, one way to assess that is by tracking student’s interest via sending a survey to them in their fifth year, then sixth, then the internship, then residency to see how their views and perception changing over time in presence of a surgical role model. Also exploring why medical students avoid surgery.

## Conclusion

Analysis of the survey results revealed two main areas that female surgical residents identified as important in their career; the important role of the surgical consultants and senior surgical residents as they need to become more aware of the example that they set for junior colleagues by provide appropriate role models as well as increase the opportunities of female to work directly with a mentor. The second thing is that the presence of female surgeons in the field unconsciously attract female to choose surgery and only by achieving a greater understanding of sex differences in general surgery can increase the proportion of female surgeons in the future.

## Data Availability

The datasets used and\or analyzed during the current study are available from the corresponding author on reasonable request.
